# Short Narrow Dental Implants versus Long Narrow Dental Implants in Fixed Prostheses: A Prospective Clinical Study

**DOI:** 10.3390/dj10030039

**Published:** 2022-03-04

**Authors:** Eduardo Antiua, Virginia Escuer, Mohammad H. Alkhraisat

**Affiliations:** 1 Clínica Eduardo Anitua, 01007 Vitoria, Spain; vir.scuer@gmail.com; 2 University Institute for Regenerative Medicine and Oral Implantology—UIRMI (UPV/EHU—Fundación Eduardo Anitua), 01007 Vitoria, Spain; 3 BTI Biotechnology Institute, 01005 Vitoria, Spain

**Keywords:** alveolar bone atrophy, narrow dental implant, short dental implant, oral surgical procedures, alveolar bone loss

## Abstract

There is a paucity of studies that assess short and narrow dental implants. This prospective study aimed to evaluate the performance of both short (≤8 mm) and narrow (≤3.5 mm width) dental implants supporting fixed prostheses in the atrophic maxilla or mandible. Towards that aim, patients with short implants were included in the study. The control group was those with long and narrow dental implants (length > 8 mm and diameter ≤ 3.5 mm). Clinical and demographic variables were extracted from clinical records. During the follow-up, implant survival and marginal bone loss were evaluated and statistically analysed. Forty-one implants were included (18 and 23 implants in the test and control groups, respectively). The median follow-up time was 26 months since insertion in both groups. The results revealed that there was no implant failure and no statistically significant differences in terms of marginal bone loss. Only one screw-loosening effect occurred in the short implants group. Short, narrow dental implants could be an alternative for the restoration of severely resorbed jaws.

## 1. Introduction

The art and science of dentistry has been evolving around the concept of keeping oral tissues healthy. This had has a positive effect, demonstrated by the decrease between 1990 and 2010 in the incidence and prevalence of severe tooth loss globally, regionally, and at the country level [1]. The prevalence of tooth loss increases with age and incidence shows a peak at 65 years. The resorption of alveolar bone as a consequence of teeth loss is characterized by being progressive, cumulative, and irreversible [2]. The rate of bone loss is the highest during the first year after tooth loss and in particular during the first months [3]. On one hand, the posterior regions are more prone to resorption, and on the other, the mandible are more vulnerable than the maxilla. In posterior segments, the alveolar ridges are mostly well-rounded but with reduced height or process with depressed bone levels [4]. Edentulism creates a situation of hypofunction and consequently decreases bone mineralization and alters blood supply [4,5,6,7]. These changes, together with the increased frequency in overaged bone, negatively affect the stability of the alveolar bone [7,8]. Edentulism not only has a local effect but may also have a negative impact on the health of the patient. For example, teeth loss is an independent risk factor for obstructive sleep apnea [9].

The treatment of edentulism revolves around the concept of osseointegration [10], a biochemical process involving several molecular mediators which, after anchoring a dental implant into the residual bone, results in the apposition of new bone directly onto the implant surface [11]. From the point of view of health economics, a systematic review has shown that dental implants are cost-effective in replacing single missing teeth than a conventional three-unit bridge [12]. In the case of edentulism, the use of dental implants has higher initial costs than mucosa-borne prosthesis. In the long-term, dental implants are cost-effective with a trend toward enhanced overall health and healthcare costs. Patients frequently present alveolar bone atrophy with affectation of both vertical and horizontal dimensions, which could hinder implant-based rehabilitation [13]. Bone augmentation procedures include guided bone regeneration, block grafts, sinus floor augmentation, distraction osteogenesis, and split ridge [14,15,16,17,18,19,20]. Pre-implant surgery increases surgical morbidity, cost, and time to restore the edentulous space.

Nowadays, narrow (≤3.5 mm diameter) or short (≤8 mm length) dental implants have been consolidated as a therapeutic alternative to treat atrophic maxillae. Despite the reduced ossointegration surface of narrow or short dental implants, its good performance has been repetitively reported in literature, reducing the overall need for bone augmentation procedures [21]. Regarding narrow dental implants, several systematic reviews with meta-analysis have proposed the absence of statistical differences in comparison to standard diameter implants [22,23,24]. This good performance has also been observed in premolar–molar regions, where the narrow implants’ survival rate was reported to be 98.6% after follow-ups ranging from 1 year to 12 years [25]. Recently, narrow diameter implants performed well after a mean follow-up time of nine years, achieving high survival rate and good bone stability [26,27]. It is worth mentioning that the implant loading protocol has not influenced the performance of narrow diameter implants [26]. The implant survival rate is 96.2% and 97.2% in the case of delayed or immediate loading, respectively.

Similarly, short dental implants have demonstrated high predictability after long-term follow-up (15 years) in retrospective cohorts [28,29,30]. Systematic reviews have also confirmed this predictability, showing no statistical differences in survival or marginal bone loss in comparison to standard diameter implants placed in vertically augmented sites [31,32,33]. Recently, the five-year performance of short implants resulted in high implant survival rate and good marginal stability [34,35]. It is notable that the implant loading protocol (delayed vs. immediate) has not influenced the performance of narrow diameter implants. The implant survival rate is 97.4% for the long implants group and 95.4% for the short implants group [35]. Immediate loading seems to not jeopardize the survival or bone stability around short implants. 

Nonetheless, some clinical situations present atrophic ridges with both vertical and horizontal resorption. The implant-based rehabilitation of these situations requires complex surgical interventions that are not always feasible due to the health status of the patient [36]. Conversely, considering the previously demonstrated predictability of short or narrow dental implants, a combination of both characteristics (both narrow and short dental implants) could be an available alternative for extremely resorbed jaws. To our knowledge, only one previous study evaluated the performance of both narrow and short dental implants in such extreme situations, showing satisfactory results after 1 and 3 years of follow-up [37]. However, it lacked a control group. Due to the scarce evidence covering this topic, the purpose of this prospective study is to describe the performance of short (≤8 mm) and narrow (≤3.5 mm) dental implants in terms of survival and marginal bone loss, in comparison to narrow long implants (length > 8 mm and diameter ≤ 3.5 mm). The null hypothesis is that narrow and short implants cannot have worse function than narrow and long implants. The specific aims of this study were the comparative descriptions of dental implant survival, marginal bone loss, and technical complications.

## 2. Materials and Methods

This study was reported according to the STROBE guidelines [38]. The study protocol for short dental implants was approved by the ethical committee of the University Hospital of Araba on 22 September 2017 (FIBEA-03-EP/17/implantes cortos), where 198 short implants were recruited. The inclusion criteria were patients with age ≥ 18 years, had short and/or long implants, and had signed the informed consent. The exclusion criteria were having implants placed more than 10 years ago or unloaded dental implants. Patients were recruited between June 2017 and December 2019. In this analysis, short (≤8 mm in length), narrow diameter implants (≤3.5 mm in width), and narrow diameter implants with a length >8 mm (test and control groups, respectively) were analysed. The test group was those with narrow and short implants. The control group was narrow and long implants. Severely resorbed edentulous space was meant to refer to an edentulous space where only narrow and short implants could be placed without performing any bone augmentation surgery. All procedures performed in studies involving human participants were conducted in accordance with the principles stated in the Declaration of Helsinki “Ethical Principles for Medical Research Involving ‘Human Subjects”, adopted by the 18th World Medical Assembly, Helsinki, Finland, June 1964, and as amended most recently by the 64th World Medical Assembly, Fontaleza, Brazil, October 2013. 

### 2.1. Surgical Procedures

Cone Beam Computer Tomography (CBCT) was performed for surgical planning, including determining the dimensions of the alveolar ridge and the bone density in the locations intended for implant placement. Surgical planning was performed with a specific software for CBCT analysis, BTI Scan (BTI Biotechnology Institute S.L, Vitoria, Spain). Prior to the implant surgery, patients received 2 g of amoxicillin and 1 g of acetaminophen. Local anaesthesia was administered using articaine hydrochloride with epinephrine (1:100,000). All surgeries were performed by the same surgeon (E.A.). After the elevation of full-thickness flap, low-speed bone drilling without irrigation was performed. Dental implants were placed following the manufacturer’s instructions (BTI Biotechnology Institute S.L, Vitoria, Spain). Immediate loading was performed if the bone type was I, II, or III and the insertion torque was higher than 25 Ncm. Otherwise, delayed implant loading was performed. 

The dental implant surface was bioactivated with fraction 2 (F2) of Plasma Rich in Growth Factors (PRGF-Endoret, BTI Biotechnology Institute S.L) [39]. Briefly, for PRGF preparation, a small volume of the patients’ own citrated-blood was obtained and centrifuged, following the manufacturer instructions (KMU15, BTI Biotechnology Institute). After the centrifugation step, the plasma column above the buffy coat was separated into two fractions, fraction 1 (F1) and fraction 2 (F2). F2 was defined as the first 2 mL of the plasma column just above the buffy coat. F1 was the rest of the plasma column above the F2.

Transmucosal abutments (BTI Biotechnology Institute) were screw-retained at the torque recommended by the manufacturer. The metallic framework of the provisional composite resin prosthesis was prepared using pre-fabricated titanium bars and cylinders. The bar was adapted to the distance between the two implants and glued to the cylinder’s knobs. After at least 4 months, a definitive porcelain fused to metal prosthesis was placed. 

### 2.2. Data Collection and Analysis

Demographic and clinical data were obtained, and implant failure was described as the non-presence of the implant at the last visit. Similarly, marginal bone loss was calculated from a radiographical record in the last available visit, taking as reference the radiograph at implant loading [40]. Marginal bone loss was measured in panoramic radiographs using a computer software (Sidexis XG, Dentsply Sirona Iberia, Barcelona, Spain). Measurements were calibrated by the length of the implant. Changes in marginal bone level were measured mesially and distally for each implant. The measurements were then averaged for each implant to calculate the marginal bone loss. The values of the marginal bone loss of all implants were then averaged and reported. A positive value indicated that the bone level was below the implant platform and a negative value indicated that the bone level was above the implant platform.

### 2.3. Statistical Analysis

Intention-to-treat analysis was performed. Normal distribution of study variables was checked with Saphiro–Wilk statistic. Quantitative variables were compared by the Mann–Whitney test and expressed in median and range. Categorical variables were expressed in frequency and compared by the Chi-square test. *p*-value < 0.05 was set for statistical significance. SPSS 15.0 for Windows statistical package software was used for statistical analysis (IBM, Armonk, NY, USA).

## 3. Results

In this study, 41 implants placed in 24 patients were included. Eighteen short and narrow and 23 narrow and long dental implants were included. Seventeen patients were females and seven were males. The median of age was 65 years (range: 42 to 72 years). Thirteen patients participated with one implant, 8 patients with 2 implants, and one patient with 6 implants. Sixteen patents participated with short narrow implants and long narrow implants.

**Figure 1:**
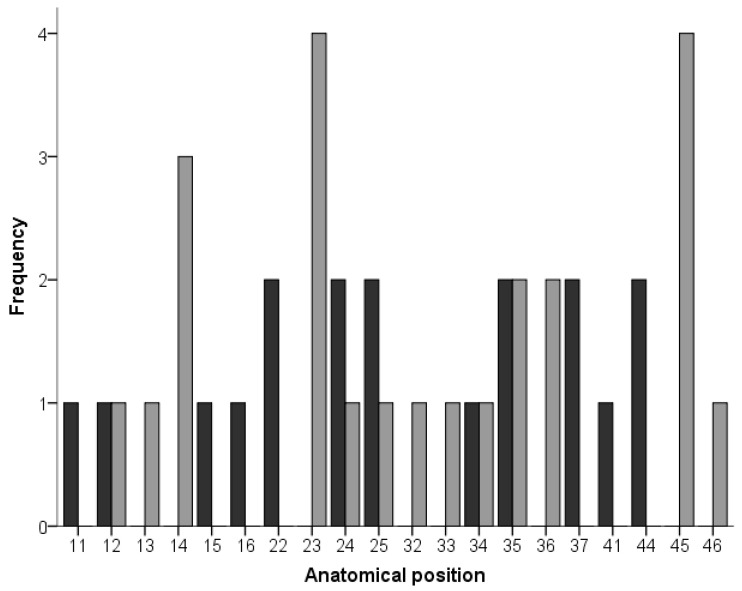
The location of the implants in the test (black) and control (grey) groups. Implant position was defined following the FDI tooth numbering system.

Table 1 shows the diameter of the implants in the test and control groups. In both groups, the most frequent diameters were 3.3 and 3.5 mm. Table 2 shows the frequency of the length of the implants in both groups. Figure 1 shows the anatomical position of the dental implants. Eighteen implants were placed in posterior sectors of the mandible and maxilla (from the second premolar to the second molar) and 23 implants were placed in the anterior sector (from the right first premolar to the left first premolar). In the test group, 8 implants were in the posterior sectors and 10 implants were in the anterior sector. In the control groups, the number of implants were 10 in the posterior sectors and 13 in the anterior sector. No statistically significant differences (Chi-square test; *p*-value = 0.087) were observed regarding implant position between the two groups. 

Table 3 summarizes the results of this study. Most of the implants in both groups were placed in good bone quality, bone type II being the most frequent. The insertion torque and bone quality allowed for immediate implant loading in more than half of the implants. The first quartile of the insertion torque was 26 Ncm and the third quartile was 50 Ncm. In the test group, the first and third quartiles of the follow-up time were 25 and 55 Ncm. In the control group the values were 30 and 45 Ncm, respectively. No statistical differences were observed between the groups in the insertion torque (Table 3).

The most common prosthesis type was partial-fixed prosthesis in both groups. All the implants supported a screw-retained prosthesis. The antagonist type was natural teeth in most of the cases. No statistical differences were observed in the registered clinical variables between the study groups (Table 3), including bone type, prosthesis type, antagonist type, insertion torque, and immediately-loaded implants. 

**Figure 2:**
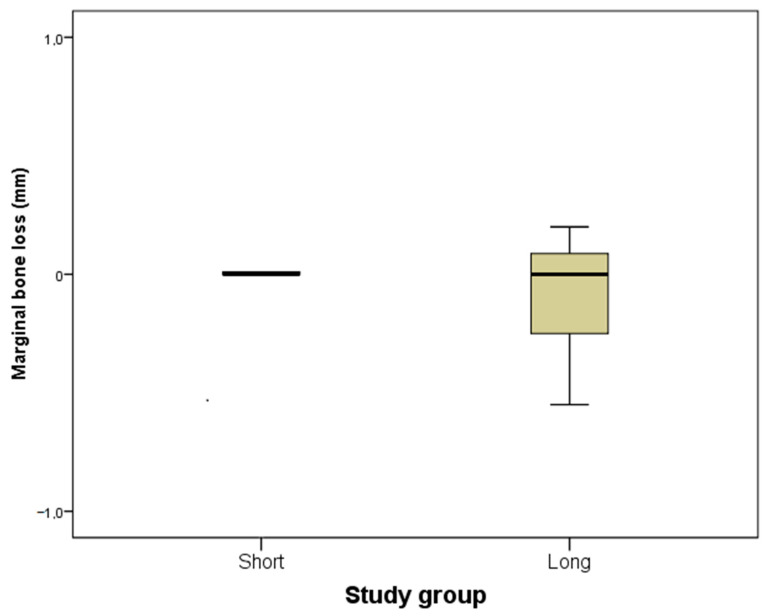
Marginal bone loss for the narrow short implants (test group) and the narrow long implants (control group).

The median follow-up time was 26 months since implant insertion in both groups. The first quartile of the follow-up time was 21 months and the third quartile was 41 months. In the test group, the first and third quartiles of the follow-up time were 18 and 37 months. In the control group, the values were 25 and 47 months, respectively. No implant failure occurred in the short and long implants. Furthermore, low marginal bone loss was observed in both groups (Figure 2). The first quartile of the marginal bone loss was −0.1 mm and the third quartile was 0.1 mm. In the test group, the first and third quartiles of the marginal bone loss were 0.0 and 0.1 mm. In the control group, the values were −0.3 and 0.1 mm, respectively. No statistical differences were observed between the groups (Table 3). Regarding technical complications, only one event of screw-loosening was observed in the short implants group. All the prosthesis survived.

## 4. Discussion

This study has shown that the performance of short (≤8 mm) and narrow (≤3.5 mm) dental implants has not resulted in statistically significant differences in terms of survival and marginal bone loss in comparison to narrow long implants (length > 8 mm and diameter ≤ 3.5 mm). Accordingly, the null hypothesis could be accepted.

The emergence of dental implants with reduced dimensions is the net outcome of several factors (material science, surface properties, and macro-design of the implant shape and connection) that converge in improving their biological and mechanical properties [41,42,43,44,45,46]. This has also been accompanied by a better understanding of the biomechanical behavior of the restoration-implant-bone under function [47,48,49,50]. Furthermore, technology is advancing to enable a precise implant placement guided by a preoperative and computer-aided treatment planning [51,52]. This is made possible by improvements in tools available for image acquisition, processing, and visualization in the CAD phase and improvement in the tools and materials in the CAM.

The dental implants’ placement in atrophic alveolar process is complicated by the proximity of the floor of the maxillary sinus and mandibular canal. Several surgical techniques have been developed to enable the placement of dental implants that vary in its degree of complexity and outcomes. Several systematic reviews have assessed several bone augmentation procedures where the type and configuration of the defect may affect the selection of the surgical technique [14,53]. Guided bone regeneration is a predictable technique that can be performed alone or in combination with other techniques [15,54]. Alveolar nerve repositioning has been described to permit osteotomy and implant placement [55,56,57]. The risk of sensory disturbances is high and could be permanent [55,56,57]. Bone block grafts need donor site and cause morbidity. Distraction osteogenesis is performed in several surgical steps and need a cooperation from the patient [58]. In this study, short (≤8 mm) and narrow (≤3.5 mm) dental implants have performed in terms of survival and marginal bone loss similar to narrow long implants (length > 8 mm and diameter ≤ 3.5 mm). Prosthodontically, cantilever extensions will save the need for alveolar bone augmentation as it avoids the placement of dental implants in the atrophic edentulous area [59,60]. Biomechanical studies have shown an increase in stress at the marginal bone level around the implant that is the closes to the cantilever extension [61,62]. However, clinical studies and systematic reviews have demonstrated the absence of significant differences in marginal bone loss between the implants near or away from the cantilever extension [59,60,63,64,65,66]. Moreover, implant and prosthesis survival is estimated to be high (>98%). [59] The design of the fixed prosthesis with cantilever extension should take into consideration all factors that can act as force modifiers (such as cantilever length, anterior–posterior spread, and parafunction). However, the ideal design should avoid the presence of cantilever extensions.

The performance of short or narrow dental implants as an alternative for the restoration of vertically or horizontally resorbed maxillae has been deeply studied and their predictability is overall well accepted by the scientific community. Recently, several systematic reviews with meta-analysis have been published reporting the good clinical performance of narrow diameter implants, strongly proposing its predictability as an alternative to bone augmentation surgeries. First, Schiegnitz and colleagues published a meta-analysis reporting high survival rates of narrow diameter implants, especially for those ranging from 3 to 3.5 mm of diameter, which presented survival rates superior to 97% and showed no statistical differences in comparison with regular diameter implants [67]. These results were in accordance with another meta-analysis published in the same year, which reported high survival rates (>95%) of narrow dental implants and low MBL after three years of follow-up [68]. Recently, two additional meta-analyses reported the absence of statistical differences between narrow and regular diameter implants in terms of survival and MBL, confirming the predictability of narrow dental implants [23,24]. No statistical differences were observed, either in terms of prosthetic survival between prostheses supported by narrow or regular diameter implants after 1 or 3 years of follow-up [24]. Prosthetic complications have been described in several studies that evaluated fixed prostheses supported by narrow diameter implants. One study reported the following complications: framework fracture (1 event), screw fractures (3 events), cementation failure (4 events), screw loosening (3 events), and ceramic fracture (1 event) [69]. Another study accounted for screw loosening (3 events), tightening of the occlusal screw (25 events), and porcelain fracture (1 event) [70]. An additional study observed 6 events of abutment screw loosening and two ceramic fractures [71]. Arisan et al. observed in 302 implants the occurrence of decementation (51 events), porcelain fracture (13 events), and screw loosening (10 events) [72]. One more study described cementation failure (4 events), abutment screw loosening (1 event), and porcelain fracture (3 events) [73]. Thus, the common technical events were cementation failure, screw loosening, and ceramic fracture. Herein, one event of screw loosening was observed in the short narrow implants group. This could be related to a failure in applying adequate torque to the screw or to mechanical overloading [74,75,76]. Moreover, technical complications have been lesser for screw-retained prosthesis than cemented prosthesis [77]. Screw-retention requires lesser interocclusal space and facilitates maintenance of the prosthesis. However, this type of prosthesis is sensitive to precision in implant placement due to the aesthetic and functional limitations of the screw emergence within the restoration [77].

Similarly, there is also strong clinical evidence on the predictability of short and extra-short dental implants as an alternative to bone augmentation techniques. A recently published meta-analysis reported similar performance of short dental implants (<8 mm) in comparison with longer implants placed in the maxilla in combination with lateral sinus floor elevation [78]. This study did not detect statistical differences between groups (implant survival, technical events, and intraoperative events). These results are in agreement with another meta-analysis, which compared the performance of short dental implants (from 5 to 8 mm length implants) with longer implants (>10 mm) placed in combination with bone augmentation surgeries [79]. Moreover, two recently published meta-analyses of randomized clinical trials also confirmed the good predictability of short dental implants. The first one compared short dental implants (<8 mm) with longer implants placed in surgically augmented sites [80], showing no statistical differences in terms of implant survival after 1 or 3 years of follow-up. Besides, lower occurrence of post-surgical complication and lower marginal bone loss was observed in short dental implants. The second one compared the performance of extra-short dental implants (<6 mm) in comparison with longer implants (>10 mm) placed in combination with sinus floor elevation [81]. After three years of follow-up, no statistical differences in terms of implant survival were observed between groups. Nevertheless, short dental implants presented lower occurrence of biological complications and lower marginal bone loss. Finally, this meta-analysis also reported that prosthetic rehabilitations supported by extra-short dental implants resulted in shorter surgical times and lower economical costs.

In this context and given the strong evidence supporting the predictability of narrow or short dental implants, it is reasonable to hypothesize that dental implants combining these characteristics (short and narrow dental implants) could be a treatment alternative for extremely resorbed maxillae. Nonetheless, there is scarce evidence reporting the performance of this kind of implants. In this retrospective study, the performance of short (≤8 mm), narrow (≤3.5 mm) dental implants in comparison to narrow dental implants with length > 8 mm has been investigated. No statistical differences were observed between groups in terms of survival or marginal bone loss. To our knowledge, there is only one prior publication reporting the outcomes of short and narrow dental implants (7 mm and 8.5 mm length and 3.3 mm width dental implants). They were shown to support single or partial rehabilitation in premolar and molar positions in both maxillae. Two out of 30 implants failed after 3 years of follow-up, providing a cumulative survival rate of 93.4% [37].

These results encourage the use of short and extra-short narrow dental implants to restore atrophic jaw although, given the scarce evidence on the performance of these dental implants, the clinical situations should be chosen with caution. In this sense, appropriate bone density [82,83] and implant splinting [84] could significantly increase the success rate and reduce the stress to the adjacent bone. The immediately loading of short and narrow implants (conditioned to bone type and insertion torque) has not negatively affected their survival or marginal bone stability. The good clinical performance of short narrow implants are thus the outcomes of the interaction of host, surgical, and implant factors. The majority of the implants were placed in good bone quality that contributed to the initial stability of the dental implants, protecting them from excessive micro-movements during the osseointegration process [35,85]. The high hydrophilicity and adapted roughness of the surface of the dental implants are factors that would enhance the biological integration of the dental implants [43,45,46]. Moreover, the use of plasma rich in growth factors has increased the bone-to-implant contact at the early stages of healing, indicating the importance of controlling the microenvironment of the dental implants during the ossoeintegration process [86].

The implant-based rehabilitation of atrophic maxillae with both vertical and horizontal component can be challenging. Several protocols have been published regarding the management of these situations. The combination of bone augmentation techniques, such as sinus floor elevation and horizontal bone grafting, has been proposed, showing good long-term results [36]. Other more invasive protocols such as calvarial bone grafts [87] or segmental osteotomy and tunnel techniques [88] have also been proposed. Among their differences, all these techniques increase the treatment time, cost, and morbidity caused to the patient. In the light of the results observed in this prospective study, the use of both narrow and short dental implants could minimize the application of these techniques, both reducing the complexity of the surgeries and maintaining good clinical outcomes.

The results here presented are subjected to several limitations. There was no randomization or blinding in this study. The small sample size is another limitation. However, it was performed according to clinical practice, which may reflect a real scenario for the use of narrow and short implants. Clinically, patients with a degree of alveolar bone loss that only allows for the insertion of narrow and short implants are not frequent in clinical practice. This explains the relatively small size of the study. Another limitation of the study is related to factors such as the inclusions of implants in both jaws, no restriction on tooth position or fixed prosthesis type, and the variability of different diameter and length of the implant. Moreover, most of the implants has been splinted to other implants by the superstructure. Although preoperative bone thickness was not recorded, the implants dimensions were selected according to the availability of bone substance. While placing of short and narrow implants was possible, wider or longer implants could not be placed without bone augmentation. A 1:1 calibration of the panoramic radiograph was performed for proximal bone measurements. This reduces error and makes them acceptable for clinical use [89]. This study could encourage the clinical performance of randomized clinical trials to assess the use of narrow and short dental implants.

## 5. Conclusions

Short and narrow dental implants could be an alternative for the restoration of severely resorbed jaws. Their clinical performance (implant survival and marginal bone stability) has been comparable to long, narrow implants. Further clinical research is needed to assess the clinical performance of narrow and short dental implants.

## Figures and Tables

**Table 1:** Diameter of the short and long implants.

Diameter (mm)	Study Groups		*p*-Value
	Short Implants	Long Implants	
2.5	0	2	0.087 ^1^
3.0	0	1	
3.3	5	9	
3.5	13	11	
	Total: 41		

^1^ Chi-square test.

**Table 2:** Length of the short and long implants.

Length (mm)	Study Groups		*p*-Value
	Short Implants	Long Implants	
6.5	5		0.000 ^1^
7.5	13		
8.5		12	
10.0		8	
11.0		1	
11.5		1	
13.0		1	
	Total: 41		

^1^ Chi-square test.

**Table 3:** Summary of the main results.

		Overall	Test Group	Control Group	*p*-Value
Number of implants		41	18	23	-
Bone type	Type I	11	7	4	0.397 ^1^
	Type II	19	7	12	
	Type III	10	4	6	
	Type IV	1	0	1	
Immediate loading (Number of implants)		25	13	12	0.192 ^1^
Insertion torque (Ncm) (Median; range)		40; 5 to 65	45; 5 to 60	40; 15 to 65	0.545 ^2^
Fixed Prosthesis Type	Single-unit	3	1	2	0.251 ^1^
	Partial	36	15	21	
	Complete	2	2	0	
Antagonist type	Tooth	28	11	17	0.170 ^1^
	Implant	11	7	4	
	Implant and tooth	2	0	2	
Time of follow-up (Months) (Median; range)		26; 4 to 114	26; 4 to 57	26; 10 to 114	0.240 ^2^
MBL (mm) (Median; range)		0.0; −1.9 to 2.8	0.0; −1.1 to 1.0	0.0; −1.9 to 2.8	0.761 ^2^

^1^: Chi-square test. ^2^: Mann–Whitney test.

## Data Availability

All the data obtained in this research are described in the manuscript.
